# Trauma-focused group intervention for unaccompanied young refugees: “Mein Weg”—predictors of treatment outcomes and sustainability of treatment effects

**DOI:** 10.1186/s13034-019-0277-0

**Published:** 2019-04-01

**Authors:** Elisa Pfeiffer, Cedric Sachser, Dunja Tutus, Joerg M. Fegert, Paul L. Plener

**Affiliations:** 1 Department of Child and Adolescent Psychiatry/Psychotherapy, University Hospital Ulm, Ulm University, Steinhoevelstraße 5, 89075 Ulm, Germany; 2 Department of Child and Adolescent Psychiatry, Medical University of Vienna, Waehringer Guertel 18-20, 1090 Vienna, Austria

**Keywords:** Predictors of the treatment outcome, PTSD, Refugees, Sustainability of treatment effects, Trauma-focused group intervention, Trauma

## Abstract

**Background:**

Current research on treatment predictors and long-term effects of trauma-focused interventions for (unaccompanied) refugee minors is limited. This secondary analysis of a recent randomised controlled trial (RCT), evaluating the trauma-focused group intervention “Mein Weg” (English “My Way”) compared to usual care, investigated several refugee-specific factors such as treatment predictors and sustainability of treatment gains.

**Methods:**

In total *N* = 50 participants (*M*_age_ = 17.00, 94% male) were included in this analysis. Evaluation of 3-month follow-up data included: posttraumatic stress symptoms [(PTSS) CATS-Self, CATS-Care], depression (PHQ-8), and dysfunctional posttraumatic cognitions (CPTCI-S). Baseline symptom severity of the above-mentioned measures, trauma load and socio-demographic factors were investigated as the treatment predictors.

**Results:**

Intention-to-treat-analyses (ITT) revealed the sustainability of treatment effects in self-reported PTSS (pre to post change: 6.48 ± 1.60, *d *= 0.62, *p *< 0.001; post to 3-month follow-up change: 1.41 ± 1.96, *d *= 0.11, *p *= 0.47) and depression (pre to post change: 7.82 ± 2.09, *d *= 0.64, *p *< 0.001; post to 3-month follow-up change: 1.35 ± 2.17, *d *= 0.05, *p *= 0.54). Country of origin alone was a significant predictor of the change in PTSS (*b *= − 8.22 ± 3.53, *t*(30) = − 2.33, *p *= 0.027), and baseline levels of depression were a significant predictor of the change in depression (*b* = 0.83 ± 0.19, *t*(33) = 4.46, *p* < 0.001).

**Conclusion:**

This group intervention can serve as a valuable component in a stepped care approach with promising long-term effects for young refugees.

*Trial registration* DRKS, #DRKS00010915. Registered 15 September 2016, https://www.drks.de/drks_web/navigate.do?navigationId=trial.HTML&TRIAL_ID=DRKS00010915

**Electronic supplementary material:**

The online version of this article (10.1186/s13034-019-0277-0) contains supplementary material, which is available to authorized users.

## Background

In 2016 alone, 63,245 unaccompanied young refugees (UYRs) applied for asylum in Europe, more than half of them (57%) in Germany [1]. UYRs experience on average eight different types of traumatic events pre-/peri- and post-migration [2–5] and often go on to develop trauma-related disorders such as posttraumatic stress disorder (PTSD), depression or anxiety. Recent studies report that 40–60% of UYRs report elevated posttraumatic stress symptoms (PTSS) [3, 4]. Levels of depression are somewhat lower, ranging from 24 to 50% [6].

There is a growing body of literature not only on traumatised refugees’ psychopathology [3, 4, 6] but also on treatment options for their symptoms [7–11]. Several individual trauma-focused interventions have proved successful in reducing PTSS in this cohort [12–14]. In order to overcome prevalent barriers to individual therapy, such as a lack of therapists, translators or financing, school- and community-based interventions have been proposed and evaluated with young refugees. In a recent review by Tyrer and Fazel [15], 21 school- and community-based interventions for refugee minors were analysed and generally found to be effective. UYRs showed a significant decrease in PTSS and depression after taking part in evidence-based group programmes such as *Teaching Recovery Techniques* (TRT) [16] or other cognitive behavioural therapy (CBT) group programs [9]. Furthermore, a review and meta-analysis of school- and community-based interventions concluded that school professionals or social workers can be successfully deployed to provide interventions for traumatised minors [17]. All of the above described interventions can be labelled as “psychosocial” interventions, which are normally administrated in a group format e.g. by social workers and take place in alternative settings such as schools or child and adolescent welfare (CAW), not in (specialized) clinics or private practice by board certified medical or psychological psychotherapists. The trauma-focused group intervention “Mein Weg” (English: “My Way”) is such a psychosocial intervention, specifically designed for UYRs and implemented by trained and supervised social workers in CAW programmes in Germany. The feasibility of the six session CBT-based group intervention as well as significant improvements in PTSS have been demonstrated in a pilot study [2]. A recent randomised controlled trial (RCT), comparing the intervention to usual care in CAW programmes with *N* = 99 UYRs, demonstrated its efficacy in decreasing PTSS and depression in this group [7].

When investigating treatment effects in UYRs, it is important to bear in mind that individual differences may affect success in mental healthcare interventions [18]. Social factors such as discrimination and changing social roles, or separation from family have been found to act as barriers to positive psychological outcomes in refugee populations [19–22]. High pre-treatment levels of depression [23, 24] and poor general mental health [25] have been found to predict poor treatment response in refugee samples. To our knowledge, the potential impact of the number of traumatic events (trauma load) and of the PTSS level pre-treatment on treatment outcomes has not been investigated in adolescent refugee samples. Furthermore, varying countries of origin involving different escape routes starting in the Middle East or in African countries have not been researched. Coming of age is a crucial time point for UYRs as this often involves a change in their legal status. At the age of 17 many of them face major uncertainty and helplessness in the asylum process. In the long run this might affect their mental health and treatment response [19]. Hence, specific peri-and post-migration factors need to be taken into account when evaluating treatment for this cohort, as post-migration stress also predicted both levels of anxiety as well as depression in a longitudinal study of UYRs [26].

Although studies on the sustainability of the treatment effects of well-established trauma-focused individual treatments such as KIDNET [27] with refugee samples are available, little is known about the long-term effects of trauma-focused treatments, especially regarding group interventions [28, 29]. This issue is, however, particularly relevant as insufficient trauma recovery is associated with academic and behavioural problems, social withdrawal and elevated anxiety or depression [30, 31]. In fact, existing findings on treatment sustainability are not only rare but also controversial [17]. Several promising group interventions based on CBT principles in schools only evaluated the intervention with young refugees post-treatment [32, 33]. A study by Goodkind et al. [34] evaluating a CBT intervention in a school setting with young refugees found that PTSS levels at the 6-month follow-up rebound to baseline. Refugee minors undergoing a six-session group CBT implemented in schools showed a significant decrease in PTSS post-treatment. However, the available follow-up data, which is restricted to eight cases, showed that treatment gains could not be maintained at the 2-month follow-up [9]. Results of an early intervention in a school setting showed stable effects at 3- and 6-month follow-up assessments [35]. A study comprising war-affected children undergoing TRT showed a significant decrease in PTSS but not in depression, not only post-treatment but also at the 3-month follow-up [36]. Generally, small to medium effect sizes were found at the 3- and 6-month follow-up when the intervention was delivered by lay counsellors [15].

In sum, the potential impact of specific post-migration factors needs to be investigated in order to optimise treatment for this cohort. More research is needed on the sustainability of treatment effects for UYRs in psychosocial interventions. In order to fill this gap in the literature, we studied predictors of the intervention outcome and the sustainability of treatment effects of the “Mein Weg” trial [7]. In research question 1, we aimed to identify, in an exploratory manner, the following possible predictors of a successful outcome of the intervention: Age, time spent in Germany, country of origin as indicator of differing escape routes (Middle East vs. African country), contact to family, trauma load, and baseline scores in PTSS, depression and dysfunctional posttraumatic cognitions (PTCs). In research question 2, we examined whether the significant improvements observed post-intervention in PTSS (primary outcome) as well as in depression, dysfunctional PTCs and caregiver-reported PTSS (secondary outcomes) are maintained at the 3-month follow-up (3MFU) post-intervention assessment. Treatment gains in all measures at 3MFU were analysed in an exploratory manner. Predictor analysis and sustainability of treatment effects were studied within the “Mein Weg” intervention arm of the aforementioned RCT study.

## Methods

### Trial design

In the original study, we applied a single-blind parallel-group RCT in seven CAW agencies in southern Germany with an allocation ratio of 1:1 (“Mein Weg” vs. usual care). The study protocol was approved by the Ethics Committee at the University of Ulm (#176/16) and registered in the German Clinical Trials Registry (#DRKS00010915). All participants were assessed at baseline, post-intervention (vs. 2 months’ usual care) and at the 3-month follow-up. More information on the trial design and randomisation is available elsewhere [7].

### Participants

The participants were recruited between November 2016 and January 2017 in the collaborating CAW agencies. Eligible participants and their legal guardians were informed about the study protocol, and written informed consent and assent were obtained. Baseline assessments were performed by trained assessors from the study centre, and follow-up assessments were performed by trained social workers in the respective agencies. Participants qualified for the study on the basis of the following inclusion criteria: Being 13–21 years of age, not undergoing alternative psychological treatment, being able to participate in daily activities at CAW agencies, reporting a history of at least one traumatic event, and at least moderate PTSS (total symptom score of ≥ 19 in the Child and Adolescent Trauma Screen (CATS-Self) [37], basic command of German language, having spent at least 6 months in Germany, prospect of continuation of the current CAW program after study inclusion for at least 3 months, no acute suicidality, and willingness and ability to attend weekly sessions.

### Intervention

The manualised trauma-focused group intervention “Mein Weg*”* comprises 6 weekly 90-min sessions with two to five participants delivered by two trained and supervised social workers in each CAW agency. The core elements of each session are depicted in a workbook. The intervention content is derived from CBT principles and comprises psychoeducation, relaxation, trauma narrative and cognitive restructuring. Several elements of the intervention, such as the narrative, could be done in the participants’ mother tongue, if they preferred to do so. For more information on the intervention see Pfeiffer et al. [7].

Within this study, the intervention was delivered by 28 social workers (11 male, *M*_*age*_ = 43.25, *SD*_*age*_ = 13.41) who had on average 16.06 years of work experience (*SD* = 11.17; *range*: 0.67–37) in CAW programs, but no experience in clinical work. All social workers delivering the intervention received a 2-day training course comprising education in trauma, trauma-related disorders and training in the intervention beforehand. Experienced clinicians provided continuous weekly consultation for the social workers. Treatment fidelity was monitored via content checklists for each session which social workers filled out after every session. Overall fidelity was high (97%). Additionally, the social workers attended weekly supervisions with trained and experienced clinicians.

### Measures

#### Child and Adolescent Trauma Screen (CATS)

The primary outcome PTSS was assessed via the *Child and Adolescent Trauma Screen (CATS*-*Self)* [37]. The CATS explores the individual trauma history with an event checklist of 15 different events and the frequency of 20 PTSS based on DSM-5 criteria [38] for PTSD on a scale ranging from 0 = “Never” to 3 = “Almost always”. The overall PTSS score is calculated by adding up all scores of the 20 DSM-5 PTSD symptoms (possible range 0–60). The internal consistency of the CATS-Self was α = 0.75 in our study sample [7]. A PTSS proxy-measure was assessed by the CATS caregiver version (CATS-Care) [37]. The proxy report was completed by the individual caregiver of each UYR within the CAW agency. Internal consistency for the caregiver report in this study was α = 0.91.

#### Patient Health Questionnaire 8

Depressive symptoms were assessed using the Patient Health Questionnaire 8 (PHQ-8) which is a short version of the PHQ-9 [39]. The 8 items are based on DSM-IV criteria [40] and refer to the frequency of the symptoms during the previous 2 weeks using a scale ranging from 0 = “Not at all” to 3 = “Nearly every day”. The overall depression score is calculated by adding up all scores (possible range 0–24). The internal consistency in our sample was α = 0.76.

#### Child Posttraumatic Cognitions Inventory Short Version

Dysfunctional PTCs were measured using the Child Posttraumatic Cognitions Inventory Short Version (CPTCI-S) [41]. The 10-item questionnaire assesses the degree of agreement on a scale ranging from 1 = “Don’t agree at all” to 4 = “Agree a lot”. The overall dysfunctional PTCs score is calculated by adding up all scores (possible range 0–40). Cronbach’s α of 0.81 in the RCT indicated good internal consistency.

All questionnaires were professionally translated (forward and backward translations) into the most common native languages of the refugee population in Germany. The assessors were only blinded at the first measurement point since randomization took place afterwards. Blinding for the follow-up assessments was not possible due to practical reasons within the CAW agencies.

### Statistical methods

Research question 1: Predictor analysis was applied to the per protocol sample and to those participants in the intervention group who completed at least five of the six intervention sessions (including the trauma narrative), and provided valid assessments of relevant outcomes pre- and post-intervention (CATS-Self, PHQ-8). To investigate possible moderators of the intervention effect, we used regression analyses with change scores in PTSS and depression as the dependent variable. Covariates in our regression models were investigated in an exploratory manner due to the small sample size. Separate models were, therefore, estimated for every predictor.

Research question 2: To investigate the sustainability of treatment effects we used three approaches: (1) mixed effect models with fixed effects of time (pre-intervention, post-intervention, 3MFUs were performed on all dependent variables (CATS-Self, CATS-Care, PHQ-8, CPTCI-S) with the ITT sample. Mixed effect models can handle missing data under the missing at random assumption. Little’s MCAR test indicated that data for all outcomes were missing completely at random for each outcome variable. Parameters were estimated using the restricted maximum likelihood (REML) method. Based on the longitudinal design of the study, data were nested by participants and repeated measures were modelled using an unstructured covariance matrix based on the comparison of likelihood criteria (AIC and BIC). (2) Additionally, a per protocol analysis was applied to those participants in the intervention group who completed at least five of the six intervention sessions and provided valid assessments of the relevant outcomes for all three time points. Given the exploratory nature of the secondary analyses, the significance level was set at *p* = 0.05 (2-tailed) for all analyses. Effect sizes (Cohen’s *d*) were calculated for pre to post, pre to 3MFU and post to 3MFU differences using the pooled standard deviation of the pre- and post-intervention score. The pooled standard deviation was used for the post to follow-up difference. (3) Finally, we calculated the reliable change index (RCI) [42] to check for clinically significant improvement or clinically significant deterioration from post-intervention to the 3MFU in order to gain an impression of treatment sustainability on a single person level. Based on the reliability *α* = 0.90 and the standard deviation of the CATS-Self measured at post-treatment, a score of 10.21 points on the scale indicated a reliable change in PTSS. Based on the reliability α = 0.82 and the standard deviation of the PHQ-8 measured at post-treatment, a score of 6.02 points on the scale indicated a reliable change.

All analyses were performed using the SPSS version 23. All data were double-entered.

## Results

### Participant flow

**Fig. 1:**
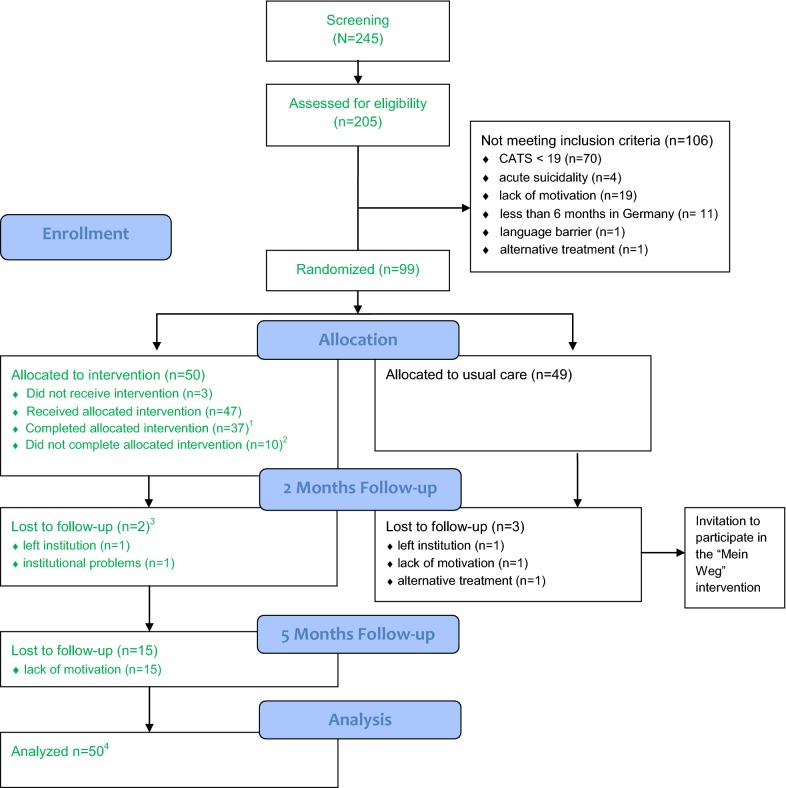
Study Flow Chart. Participants included in this study are marked in green color. ^1^Participants who started the intervention and completed at least 5 sessions of the intervention “Mein Weg”. Study sample of research question 1. ^2^ Reasons for premature termination of the intervention “Mein Weg” were lack of motivation (*n* = 4); alternative treatment (*n* = 1); high psychosocial stress due to deportation notice (*n* = 1); and organizational reasons within the institution (*n *= 4). ^3^Lost to follow-up means that participants didn’t fill out any questionnaire. ^4^Study sample of research question 2 (ITT analysis)

Altogether *N* = 50 participants within seven CAW agencies fulfilled the inclusion criteria and were allocated to the “Mein Weg” group. Demographic data on the sample are given in Table 1, the participant flow and study samples can be found in Fig. 1. For more information on the entire study sample, see the efficacy study [7]. Once assigned to the “Mein Weg” group, *n* = 47 (94%) received the allocated intervention and *n* = 37 (74%) completed the full format of at least five sessions. Altogether *n* = 2 (4%) participants did not complete the assessment at post-intervention due to relocation to another CAW agency (*n* = 1) and organisational problems within the CAW agency (*n *= 1). Drop-outs did not complete the intervention either. Hence, the post-intervention completer sample (sample for research question 1) comprised all *n* = 37 intervention completers. There were no statistically significant differences between the completer and non-completer samples in terms of age, gender, country of origin, duration of stay in Germany, trauma load or baseline scores.

**Table 1:** Sample description at baseline of the “Mein Weg” (engl. My Way) group (*N* = 50)

	*n*	*M* (*SD*) or %	*Range*
Age (years)	50	17.00 (*1.11*)	14–19
Gender			
Male	47	94.0	
Female	3	6.0	
Country of origin			
Middle East country	35	70.0	
African country	15	30.0	
Duration of stay (months)			
In Germany	47	12.66 (*4.01*)	6–26
In the institution	49	9.44 (*3.92*)	3–20
Family contact (%)			
No contact	13	26.5	
Daily	3	6.1	
Weekly	18	36.7	
Monthly	11	22.4	
Several times a year	4	8.2	
Number of traumatic events	35	8.63 (*2.81*)	2–13

At the 3MFU assessment post-intervention *n* = 17 participants (34%) in the “*Mein Weg”* group were lost due to lack of motivation to fill out more questionnaires. The sample for research question 2 comprised all participants (*N*= 50) in the ITT analysis and a subsample (*n* = 22 to *n* = 24; depending on the measure) in the 3MFU completer analysis.

### Research question 1: predictor analysis

Separate models were estimated for all eight predictors within the completer sample for PTSS and depressive symptoms as the dependent variable. With regard to PTSS only the factor country of origin, which was dichotomised to countries in the Middle East (*n* = 23, mean change = 4.22) vs. African countries (*n* = 9, mean change 12.44), was found to statistically significant predict treatment response. The effect was indicated by an 8.22 point (*d* = 0.95, *p* = 0.027) higher mean change on the CATS-Self scale for participants from African countries compared to participants from countries in the Middle East. Thereby it seems noteworthy that the number of different trauma types and rates of endorsement of different trauma types were comparable among both subgroups (Middle East vs. Africa). With regard to depressive symptoms, only the factor severity of depression pre-intervention was found to statistically significant predict treatment response. The effect was indicated by a higher treatment response by a 0.83 point (*d* = 0.30; *p* < 0.001) higher mean change in depression for participants with higher levels of depressive symptoms prior intervention (see Table 2).

**Table 2:** Predictors of treatment response for the depended variables posttraumatic stress symptoms (PTSS) and depressive symptoms in the per protocol sample

Predictor	Predictor models PTSS		Predictor models depressive symptoms	
	Estimate b ± SE b 95% CI	Statistic	Estimate b ± SE b 95% CI	Statistic
Age (years)	− 0.07 ± 1.40 − 2.93, 2.78	*t*(30) = − 0.05 *p* = 0.960	− 0.39 ± 0.90 − 2.21, 1.45	*t*(33) = − 0.43 *p* = 0.672
Time in Germany (months)	0.20 ± 0.43 − 0.68, 1.07	*t*(29) = 0.46 *p* = 0.650	0.34 ± 0.28 − 0.22, 0.90	*t*(32) = 1.24 *p* = 0.225
Country of origin (Middle East vs. Africa)	− 8.22 ± 3.53 − 15.44, − 1.01	*t*(30) = − 2.33 *p* = 0.027	− 1.24 ± 2.51 − 6.33, 3.86	*t*(33) = − 0.49 *p* = 0.625
Contact to family (no/yes)	− 1.32 ± 3.61 − 8.71, 6.07	*t*(29) = − 0.37 *p* = 0.718	− 0.09 ± 2.36 − 4.72, 4.90	*t*(32) = 0.04 *p* = 0.970
Traumaload (number of events)	0.96 ± 0.77 − 0.64, 2.57	*t(20)* = 1.25 *p* = 0.224	− 0.15 ± 0.52 − 1.23, 0.93	*t*(20) = − 0.30 *p* = 0.771
Baseline severity posttraumatic stress symptoms	0.16 ± 0.23 − 0.31, 0.62	*t*(30) = 0.69 *p* = 0.497	0.12 ± 0.14 − 0.17, 0.41	*t*(32) = 0.85 *p* = 0.402
Baseline severity depressive symptoms	− 0.02 ± 0.37 − 0.77, 0.74	*t*(30) = − 0.04 *p* = 0.965	0.83 ± 0.19 0.45, 1.21	*t*(33) = 4.46 *p* < 0.001
Baseline severity dysfunctional cognitions	− 0.03 ± 0.29 − 0.62, 0.56	*t*(30) = − 0.10 *p* = 0.920	− 0.16 ± 0.18 − 0.52, 0.20	*t*(33) = − 0.90 *p* = 0.377

Separate models were calculated for every predictor

### Research question 2: sustainability of treatment effects

A post hoc power analysis to detect a difference between the two depended means [*n* = 50, alpha level 0.05 (two tailed, statistical power of 0.80)] indicated that a statistically significant mean difference (improvement or deterioration) was found for effects higher than *d* = 0.40.

From post-intervention to 3 MFU no statistically significant mean improvement or deterioration was described for self-reported symptoms of PTSS, depression or dysfunctional PTCs (see Table 3 and Additional file 1: Tables S1–S3). Improvements due to participation in the “Mein Weg” intervention on PTSS and depression were stable in the FU period as indicated by comparable pre-post and pre-3MFU effect sizes. Dysfunctional PTCs deteriorated between post-intervention and 3MFU but were still lower compared to pre-intervention.

**Table 3:** ITT: treatment outcomes: estimated marginal means (M), standard errors (SE), 95% confidence intervals (95% CI) for Pre-, posttreatment and 3-month follow-up (3MFU)

	Pre-intervention	Post-intervention	3MFU	Difference: pre–post		Difference: pre-3MFU		Difference: post-3MFU	
	*M *±* SE* 95% *CI*	*M *±* SE* 95% *CI*	*M *±* SE* 95% *CI*	*M *±* SE* 95% *CI*	Statistics	*M *±* SE* 95% *CI*	Statistics	*M *±* SE* 95% *CI*	Statistics
CATS-Self	29.91 ± 1.16 27.58, 32.25	23.44 ± 1.81 19.79, 27.08	22.09 ± 2.27 17.46, 26.72	6.48 ± 1.60 3.24, 9.71	*p* < 0.001 *d *= 0.62	7.82 ± 2.09 3.55, 12.10	*p* < 0.001 *d* = 0.64	1.35 ± 2.17 − 3.09, 5.78	*p* = 0.539 *d* = 0.05
CATS-Care	18.47 ± 1.56 15.33, 21.60	18.43 ± 1.38 15.64, 21.21	18.00 ± 1.66 14.65, 21.36	0.04 ± 1.53 − 3.04, 3.12	*p* = 0.979 *d *= 0.00	0.46 ± 1.62 − 2.83, 3.75	*p* = 0.778 *d *= 0.04	0.42 ± 1.51 − 2.64, 3.49	*p* = 0.781 *d *= 0.04
PHQ-8	11.52 ± 0.71 10.08, 12.95	8.28 ± 0.77 6.73, 9.83	8.17 ± 0.95 6.24, 10.10	3.24 ± 0.87 1.50, 4.99	*p* = 0.001 *d *= 0.62	3.35 ± 1.02 1.28, 5.43	*p* = 0.003 *d *= 0.57	0.11 ± 0.90 − 1.73, 1.94	*p* = 0.905 *d *= 0.02
CPTCI-S	13.18 ± 0.91 11.35, 15.00	9.06 ± 1.06 6.92, 11.20	10.80 ± 1.28 8.21, 13.39	4.11 ± 1.04 2.01, 6.22	*p* < 0.001 *d *= 0.59	2.38 ± 1.04 0.25, 4.51	*p* = 0.030 *d *= 0.31	− 1.74 ± 1.23 − 4.23, 0.76	*p* = 0.166 *d *= − 0.21

Note: *N* = 50. *CATS-Self* Child and Adolescent Trauma Sreen (self-report); *CATS-Care* Child and Adolescent Trauma Screen (caregiver report); *PHQ-8* Patient Health Questionnaire 8; *CPTCI-S* Child Post-traumatic Cognitions Inventory Short Version

To investigate sustainability on a single person level we used the RCI to detect possible clinically significant improvements or deterioration within the completer sample (*n* = 24). With regard to PTSS as measured by the CATS-Self, *n* = 15 (62.5%) participants remained in a stable condition, *n* = 5 (20.8%) showed a clinically significant improvement and *n* = 4 (16.7%) showed a clinically significant deterioration according to the RCI. With regard to depressive symptoms, as measured by the PHQ-8, *n* = 20 (83.3%) participants remained in a stable condition, *n* = 2 (8.3%) showed a clinically significant improvement and *n* = 2 (8.3%) showed a clinically significant deterioration.

## Discussion

Since our RCT demonstrated the efficacy of the trauma-focused group intervention “Mein Weg*”* for UYRs, compared with usual care [7], we conducted this secondary analysis with a view to investigating treatment outcome predictors on the one hand and the sustainability of treatment effects on the other. Country of origin (Middle East vs. African countries) remained the sole significant predictor of symptom improvement in PTSS. This is surprising as numerous studies showed that social and interpersonal factors, as well as post-migration stressors and psychopathological burden affect mental health outcomes in refugees [3, 19, 43]. The finding that contact to family does not seem to have any predictive value for treatment response is somewhat counterintuitive as social support plays an important role in trauma recovery. Future research needs to address not only the quantity but also the quality of the contact in order to derive conclusions for interventions delivered to this cohort. The finding that UYRs from countries in the Middle East benefit less from the intervention might be explained by the general increase in the number of deportation notices among Afghan youth [44]. Refusal of asylum was closely associated with higher levels of psychological distress in UYRs in Norway [45]. Afghan UYRs in particular are afraid of being deported as Afghanistan has been declared a “safe country of origin” by the German government. As this study sample mainly comprised Afghan youth in the Middle East group (*n* = 19), this threat might have overshadowed their benefit from the intervention. In fact, further analysis revealed that coming from Afghanistan was a significant predictor of poor treatment response not only in PTSS but also in depression. In fact, a necessary pre-requisite for trauma-focused treatment is the existence of a “safe place”, meaning reliable protection from ongoing traumatization. Having such a “safe place” cannot be assumed for Afghan refugees, being continuously threatened by a potential return to their previous traumatizing environment. It is therefore questionable whether refugee minors from Afghanistan can benefit from exposure-based treatments as long as they are seriously threatened by deportation.

The symptom improvement in depression was only predicted by higher baseline scores in depression. This is in line with a longitudinal multilevel analysis of a study with refugees and asylum-seekers suffering from PTSD undergoing eye movement desensitisation and reprocessing (EMDR) and stabilisation [24]. This finding shows that highly affected UYRs who may fulfil all the criteria for a depressive disorder (and probably also PTSD) benefit from psychosocial interventions. The finding might also be explained by the fact that the study was not powered for symptom reduction in depression. Furthermore, mean severity at baseline was only moderate with 11.52 points on a possible range 0–24, so for some participants with low symptoms in depression, there was less room for improvement.

In sum, the results of the predictor analysis are promising as many different participants might benefit equally from the intervention independently of age or psychopathology. However many questions remain as findings from our study contradict earlier studies on the influence of predictors for treatment response. This might be due to the limited number of participants.

The results of the sustainability analyses demonstrated that treatment gains in self-reported PTSS and depression remained clinically stable on a mean level and single person level over the course of the 3 months post-intervention. Especially since literature on the long-time effects of psychosocial interventions is scarce and controversial, this is an important finding that backs similar evidence in (early) psychosocial interventions [15, 35, 36]. A trend was found that dysfunctional PTCs deteriorated between post-intervention and 3MFU but were still lower compared to pre-intervention. This trend may be explained by enduring/ongoing daily stressors in the follow-up period, which may affect cognitions such as “I don’t trust people”; “I am no good”, or “I can’t cope when things get tough”.

### Limitations

The sample size of this secondary analysis was relatively small with a strong gender imbalance which greatly limits the impact and generalisability of the findings. However, most studies on psychosocial interventions with refugees include similar or smaller sample sizes [9, 46]. This highlights the need for larger RCTs to evaluate the effectiveness of these interventions. The small sample size, especially in completer samples, led to the employment of an explorative analysis approach that only included single predictor models for predictor analysis. Studies with larger samples should investigate factors that might influence treatment outcome within one model in order to evaluate confounding effects. Future studies might also include more heterogeneous samples. This study mainly comprises UYRs from Afghanistan (*n* = 19). Hence, results might not be identical for youth coming from other countries. Due to the inclusion criteria of the study, a large number of equally or potentially even more needy young refugees were excluded from the study. In a subsequent “dissemination and implementation” study conducted by the developers of the manual, these youth were invited to participate as well. Throughout this subsequent study no serious adverse events were reported. Hence, “Mein Weg” can be seen as safe and feasible for a sample of UYR without pre-selected criteria. The assessors were not blinded at the post-intervention and 3MFU assessments, which could have led to a performance and ascertainment bias after randomization. Longer follow-up assessments were not included in the study design because, when the study was being conducted, UYRs were often reassigned between CAW agencies or left the CAW programme altogether when they became of age. Since there are no follow-up data on 36% of the participants, we cannot draw any conclusions about whether they improved or deteriorated post-intervention. The measures used in this study were developed in western countries and not validated in refugee populations. Hence, we cannot assume that these measures are really appropriate for all the cultures of our study participants. The measures have revealed satisfactory psychometric properties, though. No clinical interviews by independent assessors were employed to assess the symptoms and to establish a possible diagnosis. However, a meta-analysis of trauma-focused therapy for (adult) refugees found no significant difference in effect size based on the method used to assess PTSD symptoms (clinical interview vs. questionnaire) [47].

### Future research

Alongside various other authors, Horlings and Hein [48] argue that layered systems for Europe’s mental healthcare are promising options for catering for the diverse needs of refugee minors. Theses stepped care approaches include, in addition to early psychosocial interventions, focused non-specialised interventions and they recommend short-time group interventions for refugee minors suffering from PTSD. However, as described in the introduction, little research on school-and community based interventions for traumatised refugee minors has been conducted and evaluated over a longer time period than post-treatment. As far as we know, no such research has been undertaken in Germany. A recent systematic review of school-based socio-emotional interventions for this cohort did not find a single study carried out in Germany [49]. As Germany has welcomed the highest number of UYRs in the European Union [1], there is an urgent need for more research in this field [49]. More specifically, current research should focus to a greater degree on innovative and culture-sensitive interventions in naturalistic settings. The present study on the “Mein Weg” trial can be seen as one example of how to implement psychosocial interventions with long-term effects in diverse naturalistic settings such as German CAW programmes. Additionally, (therapeutic) interventions with a more “inclusive” approach need to be considered in order to fulfil the needs of UYRs who are not stable enough for a trauma-focused intervention in a group setting.

In future research, several other, and potentially more relevant, pre- and post-migration factors such as perceived discrimination [50] or asylum status in particular [51] need to be investigated, especially with regard to the long-term effectiveness of an intervention. Apart from focused psychosocial interventions, several other layers of stepped-care approaches such as family or peer support groups or language training need to be systematically investigated, not only with regard to psychopathology but also to functional level and integration outcomes.

## Conclusions

This study increases understanding of the effectiveness of psychosocial interventions for young refugees in naturalistic settings. The intervention "Mein Weg" was found to be effective not only post-intervention but also for a further 3 months in self-reported PTSS and depression. The current study extended prior knowledge on the effect of pre-/peri- and post-migration factors on symptom reduction and hopefully stipulates more research on dismantling studies in psychosocial interventions. On a political level, the psychological consequences of an insecure asylum status need to be discussed. This intervention could be a valuable component in a stepped and collaborative care approach for UYRs in Germany. However, there is a need for more systematic research on different levels of stepped-care approaches in order to fill the ongoing gap between a large number of highly traumatised and psychologically impaired refugees, and an overstrained mental healthcare system.

## Additional file

**Additional file 1: Table S1.** Per Protocol: Treatment Outcomes: Estimated Marginal Means (M), Standard Errors (SE), 95% Confidence Intervals (95% CI) for Pre-, Postintervention and 3-month Follow-Up (3MFU). **Table S2.** Results of the Mixed Effects Models (ITT Analyses, *n* = 50). **Table S3.** Results of the Mixed Effects Models (Per protocol Analyses).
