# Supplementary material for: Trauma-focused group intervention for unaccompanied young refugees: “Mein Weg”—predictors of treatment outcomes and sustainability of treatment effects
Source: Child Adolesc Psychiatry Ment Health. 2019 Apr 1;13:18. doi: 10.1186/s13034-019-0277-0 (PMC6442414; doi:10.1186/s13034-019-0277-0)
Supplement: Supplementary file 1 — Additional file 1: Table S1. Per Protocol: Treatment Outcomes: Estimated Marginal Means (M), Standard Errors (SE), 95% Confidence Intervals (95% CI) for Pre-, Postintervention and 3-month Follow-Up (3MFU). Table S2. Results of the Mixed Effects Models (ITT Analyses, n = 50). Table S3. Results of the Mixed Effects Models (Per protocol Analyses). [file 13034_2019_277_MOESM1_ESM.docx]

| Table S1. Per Protocol: Treatment Outcomes: Estimated Marginal Means (M), Standard Errors (SE), 95% Confidence Intervals (95% CI) for Pre-, Postintervention and 3-month Follow-Up (3MFU). | | | | | | | | | | | | | | |
| --- | --- | --- | --- | --- | --- | --- | --- | --- | --- | --- | --- | --- | --- | --- |
|  | Pre-intervention |  | Post-intervention |  | 3MFU |  | Difference: Pre-Post | |  | Difference: Pre-3MFU | |  | Difference: Post-3MFU | |
|  | *M ± SE*  95% *CI* |  | *M ± SE*  95% *CI* |  | *M ± SE*  95% *CI* |  | *M ± SE*  95% *CI* | Statistics |  | *M ± SE*  95% *CI* | Statistics |  | *M ± SE*  95% *CI* | Statistics |
| CATS-Self  (*n* = 23) | 29.39 ± 1.64  25.99, 32.79 |  | 22.83 ± 2.38  17.89, 27.76 |  | 21.70 ± 2.73  16.05, 27.35 |  | 6.57 ± 2.08  2.26, 10.87 | *p* = .004  *d* = 0.68 |  | 7.70 ± 2.31  2.90, 12.49 | *p* = .003  *d* = 0.73 |  | 1.13 ± 2.43  -3.92, 6.18 | *p* = .647  *d* = 0.09 |
| CATS-Care  (*n* = 22) | 17.68 ± 2.60  12.27, 23.09 |  | 19.55 ± 2.21  14.95, 24.14 |  | 16.59 ± 2.30  11.81, 21.37 |  | -1.86 ± 2.40  -6.85, 3.12 | *p* = .446  *d* = -0.17 |  | 1.09 ± 1.09  -2.97, 5.15 | *p* = .583  *d* = 0.09 |  | 2.96 ± 1.73  -.64, 6.55 | *p* = .102  *d* = 0.28 |
| PHQ-8  (*n* = 24) | 11.50 ± .84  9.76, 13.24 |  | 8.83 ± 1.08  6.56, 11.07 |  | 8.54 ± 1.07  6.33, 10.75 |  | 2.67 ± 1.18  .23, 5.10 | *p* = .033  *d* = 0.57 |  | 2.96 ± 1.19  .49, 5.43 | *p* = .021  *d* = 0.63 |  | .292 ± 1.01  -1.81, 2.39 | *p* = .776  *d* = 0.06 |
| CPTCI-S  (*n* = 24) | 13.75 ± 1.44  10.78, 16.72 |  | 9.29 ± 1.53  6.12, 12.46 |  | 11.04 ± 1.67  7.58, 14.51 |  | 4.46 ± 1.51  1.34, 7.58 | *p* = .007  *d* = 0.61 |  | 2.71 ± 1.04  .55, 4.87 | *p* = .016  *d* = 0.36 |  | -1.75 ± 1.50  -4.85, 1.35 | *p* = .255  *d* = -0.22 |
| *Note.*  CATS-Self = Child and Adolescent Trauma Screen (self-report); CATS-Care = Child and Adolescent Trauma Screen (caregiver report); PHQ-8=Patient Health Questionnaire; CPTCI-S=Child Post-Traumatic Cognitions Inventory Short Version. | | | | | | | | | | | | | | |

| Table S2. Results of the Mixed Effects Models (ITT Analyses, *n* = 50). | | | | |
| --- | --- | --- | --- | --- |
|  | Estimates of Fixed Effects (*ITT*) | | | |
| *Outcome* | Estimate (b) | *SE*  (b) | 95% *CI* | *p* |
| CATS-Self |  |  |  |  |
| Intercept | 22.09 | 2.27 | 17.46, 26.72 | < .001 |
| Time 1 | 7.82 | 2.09 | 3.54, 12.10 | .001 |
| Time 2 | 1.35 | 2.17 | -3.09, 5.78 | .539 |
| CATS-Care |  |  |  |  |
| Intercept | 18.00 | 1.66 | 14.65, 21.36 | < .001 |
| Time 1 | .46 | 1.62 | -2.82, 3.75 | .778 |
| Time 2 | .42 | 1.51 | -2.64, 3.49 | .781 |
| PHQ-8 |  |  |  |  |
| Intercept | 8.17 | .95 | 6.24, 10.10 | < .001 |
| Time 1 | 3.35 | 1.02 | 1.27, 5.43 | .003 |
| Time 2 | .11 | .90 | -1.73, 1.94 | .905 |
| CPTCI-S |  |  |  |  |
| Intercept | 10.80 | 1.28 | 8.21, 13.39 | < .001 |
| Time 1 | 2.38 | 1.04 | .25, 4.51 | .030 |
| Time 2 | -1.74 | 1.23 | -4.23, .76 | .166 |
| *Note.* CATS-Self = Child and Adolescent Trauma Screen (self-report); CATS-Care = Child and Adolescent Trauma Screen (caregiver report); PHQ-8 = Patient Health Questionnaire; CPTCI-S = Child Post-Traumatic Cognitions Inventory Short Version. | | | | |

| Table S3. Results of the Mixed Effects Models (Per protocol Analyses). | | | | |
| --- | --- | --- | --- | --- |
|  | Estimates of Fixed Effects (*ITT*) | | | |
| *Outcome* | Estimate (b) | *SE*  (b) | 95% *CI* | *p* |
| CATS-S (*n* = 23) |  |  |  |  |
| Intercept | 21.70 | 2.72 | 16.04, 27.35 | < .001 |
| Time 1 | 7.70 | 2.31 | 2.90, 12.49 | .003 |
| Time 2 | 1.13 | 2.43 | -3.92, 6.18 | .647 |
| CATS-C (*n* = 22) |  |  |  |  |
| Intercept | 16.59 | 2.30 | 11.81, 21.37 | < .001 |
| Time 1 | 1.09 | 1.95 | -2.97, 5.15 | .583 |
| Time 2 | 2.95 | 1.73 | -.64, 6.55 | .102 |
| PHQ-8 (*n* = 24) |  |  |  |  |
| Intercept | 8.54 | 1.07 | 6.33, 10.75 | < .001 |
| Time 1 | 2.96 | 1.19 | .49, 5.43 | .021 |
| Time 2 | .29 | 1.01 | -1.80, 2.39 | .776 |
| CPTCI-S (*n* = 24) |  |  |  |  |
| Intercept | 11.04 | 1.67 | 7.58, 14.50 | < .001 |
| Time 1 | 2.71 | 1.04 | .55, 4.87 | .016 |
| Time 2 | -1.75 | 1.50 | -4.85 | 1.35 |
| *Note.* CATS-S = Child and Adolescent Trauma Screen (self-report); CATS-C = Child and Adolescent Trauma Screen (caregiver report); PHQ-8 = Patient Health Questionnaire; CPTCI-S = Child Post-Traumatic Cognitions Inventory Short Version. | | | | |
